# Cryptococcal fungemia and probable histoplasmosis in a patient infected with HIV. Case report

**DOI:** 10.1186/s12879-018-3622-7

**Published:** 2018-12-27

**Authors:** Deving Arias Ramos, John Alexander Alzate, Jhon Alejandro Rico Gallego, Natalia Acevedo Escalante

**Affiliations:** 1 Resident of internal medicine, Universidad Tecnológica de Pereira, Pereira, Colombia; 2 Internal Medicine specialist at the Hospital Universitario San Jorge, and associate professor at the Universidad Tecnológica de Pereira, Pereira, Colombia; 3 Physician. Universidad Tecnológica de Pereira, Pereira, Colombia; 4 Medical student, Universidad Tecnológica de Pereira, Pereira, Colombia; 5 Grupo de Investigación de Medicina Interna, Universidad Tecnológica de Pereira, Pereira, Colombia

**Keywords:** Cryptococcosis, Histoplasmosis, Cryptosporidiosis, Human immunodeficiency virus (HIV), Opportunistic infection

## Abstract

**Background:**

Those infected by human immunodeficiency virus (HIV) have a higher risk of opportunistic infections. The risk is related to the level of immunosuppression. We report a case of a young male with the unusual scenario of three opportunistic infections occurring simultaneously: Cryptococcosis, Histoplasmosis and Cryptosporidiosis. Histoplasmosis and cryptococcosis are major causes of morbimortality in immunocompromised patients due to HIV infection.

**Case presentation:**

We report the case of a patient with HIV infection with a CD4 T lymphocyte cell (CD4) count of 2 cells/mm3, who presented with 6 months of diarrhea, non-productive dry cough, nocturnal diaphoresis, fever, weight loss, and a maculopapular rash. He had a concurrent infection with three opportunistic microorganisms: fungemia by cryptococcosis, disseminated histoplasmosis confirmed by detection of the antigen in urine and chronic diarrhea by cryptosporidiosis confirmed by direct observation in feces by modified Ziehl–Neelsen stain. The patient received antifungal treatment with a satisfactory outcome.

**Conclusions:**

There are still regions where HIV detection programs are deficient thus facilitating occurrence of HIV infection cases in advanced stages of immunosuppression. A high level of suspicion of systemic mycoses and concurrent infection by several opportunistic pathogens is required in severely immunocompromised patients.

## Background

The most frequent systemic mycoses in immunocompromised patients are pneumocystosis, cryptococcosis, histoplasmosis, candidiasis and aspergillosis. Their incidence has been reduced in 20–25% since the introduction of highly active anti-retroviral therapy (HAART) more than two decades ago [1]. Histoplasmosis and cryptococcosis are major causes of morbimortality in immunocompromised patients due to HIV infection [2], with a global mortality of 37.2 and 81.8% respectively [1, 3]. We report the case of a patient with HIV infection with a CD4 T lymphocyte cell (CD4) count of 2 cells/mm3, who presented concurrent infection of three opportunistic pathogens: Cryptococcosis, disseminated histoplasmosis and cryptosporidiosis.

*Histoplasma capsulatum* and *Cryptococcus neoformans* are present in soils contaminated with droppings of birds, bats and pigeons [3]. The infection is acquired by inhalation of spores. *Histoplasma capsulatum* is a dimorphic fungus endemic to North America close to the Ohio and Mississippi River Valleys, Central and South America. The disease develops by initial contact with the fungus or by endogenous reactivation from a previous exposure. In immunocompromised patients it produces a progressive and disseminated disease with multi-organ involvement, mostly extrapulmonary, of the reticuloendothelial system, bone marrow and skin [4]. Cutaneous lesions have several forms: papular, macular, maculopapular or nodular eruption; flat, crusted, acneiform, purple or hemorrhagic lesions; sometimes they are ulcerative and lepromatous-like [3]. Pulmonary involvement can be seen in chest X-ray in the form of diffuse reticulonodular opacities in 70% of cases [4]. *Cryptococcus neoformans var. neoformans* is a ubiquitous saprophytic fungus, which causes an invasive infection with preference for the central nervous system in the form of meningoencephalitis. Its prevalence was reduced in western countries to less than 10% [5]. Cryptosporidiosis is caused by the protozoan parasite of the species *Cryptosporidium spp*. It is a frequent cause of diarrhea in immunocompromised patients; it can cause chronic, transient or acute profuse diarrhea. Subclinical infections are also possible [6].

## Case report

**Fig. 1:**
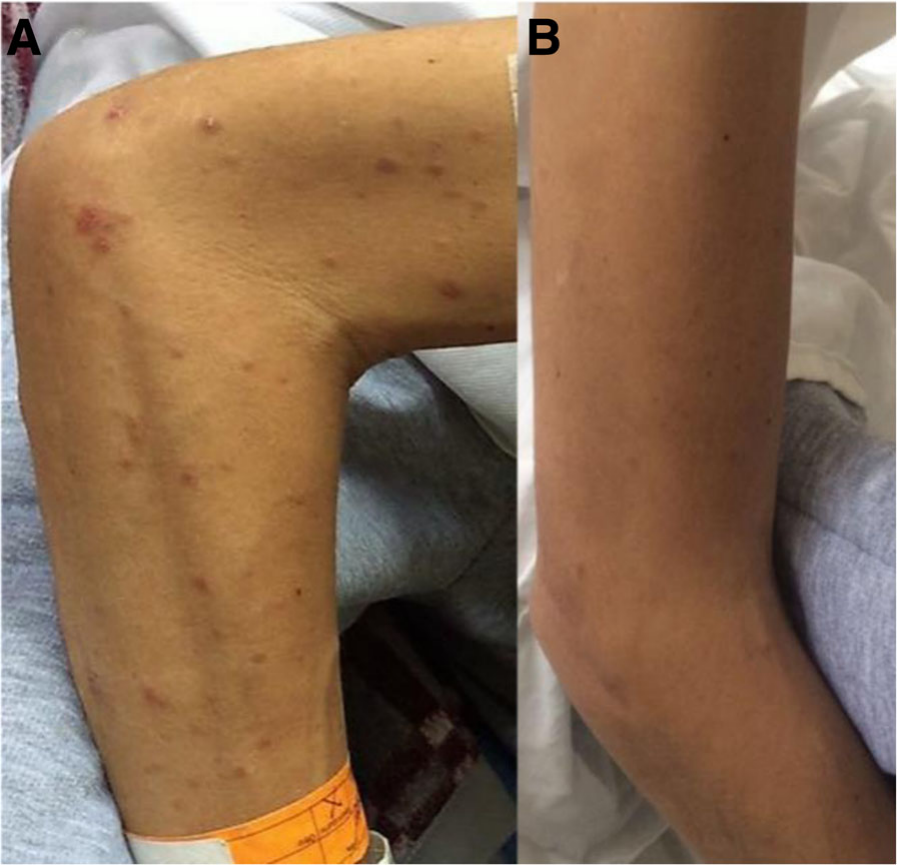
Skin of the patient’s arm before and after antifungal treatment **a**. Erythematous papules in right upper limb. **b**. The disappearance of lesions 2 weeks after treatment

A 32-year-old Colombian male, resident of an urban area, heterosexual without a stable partner. His medical record was notable for transfusion of fresh frozen plasma 4 years ago due to a consumption coagulopathy caused by sepsis secondary to perforated appendicitis. The patient consulted for 6 months of diarrhea with abundant 4 to 5 foamy, foul-smelling stools per day, no blood or mucus were present in feces. It was associated with asthenia, anorexia, and non-productive dry cough, nocturnal diaphoresis, fever and weight loss of 12 Kg (body mass index, BMI, 15.22 Kg/m2). In the last month, he presented a reddish generalized non-confluent maculopapular rash without compromise of palms and soles (Fig. 1). Physical examination showed painless hepatomegaly, without other abnormalities.

A fourth-generation human immunodeficiency virus (HIV) assay was positive, and viral load was 636.000 VIH-1 RNA copies/ml, Log10: 5.80, CD4 T lymphocyte cell (CD4) count was 2 cells/mm3. Modified Ziehl–Neelsen stain in feces showed oocysts of *Cryptosporidium spp,* and a *Multiplex Polymerase chain reaction (PCR) FilmArray* test in feces identified *Cryptosporidium* and enteropathogenic *Escherichia coli.* Esophagogastroduodenoscopy and colonoscopy were performed and did not show macroscopic abnormalities; several biopsies were taken from the third portion of the duodenum, cecum, transverse and sigmoid colon; they did not reveal any abnormalities either. Serum Cytomegalovirus (CMV) viral load was negative. The patient received treatment with Nitazoxanide 500 mg PO B.I.D for three days with resolution of the diarrhea.

On admission blood cultures were taken. Fungi grew in blood cultures. A preliminary Gram stain exhibited budding yeasts, India ink test was positive for *Cryptococcus spp*. The blood culture final report confirmed *Cryptococcus neoformans*. Lumbar puncture was performed to rule out central nervous system involvement. Cerebrospinal fluid tests were taken: cytology, chemistry, routine stains, and *Multiplex PCR FilmArray* were negative. Induction treatment with amphotericin B at a dose of 1.0 mg/kg Q.D and fluconazole 800 mg Q.D were given for 2 weeks.

**Fig. 2:**
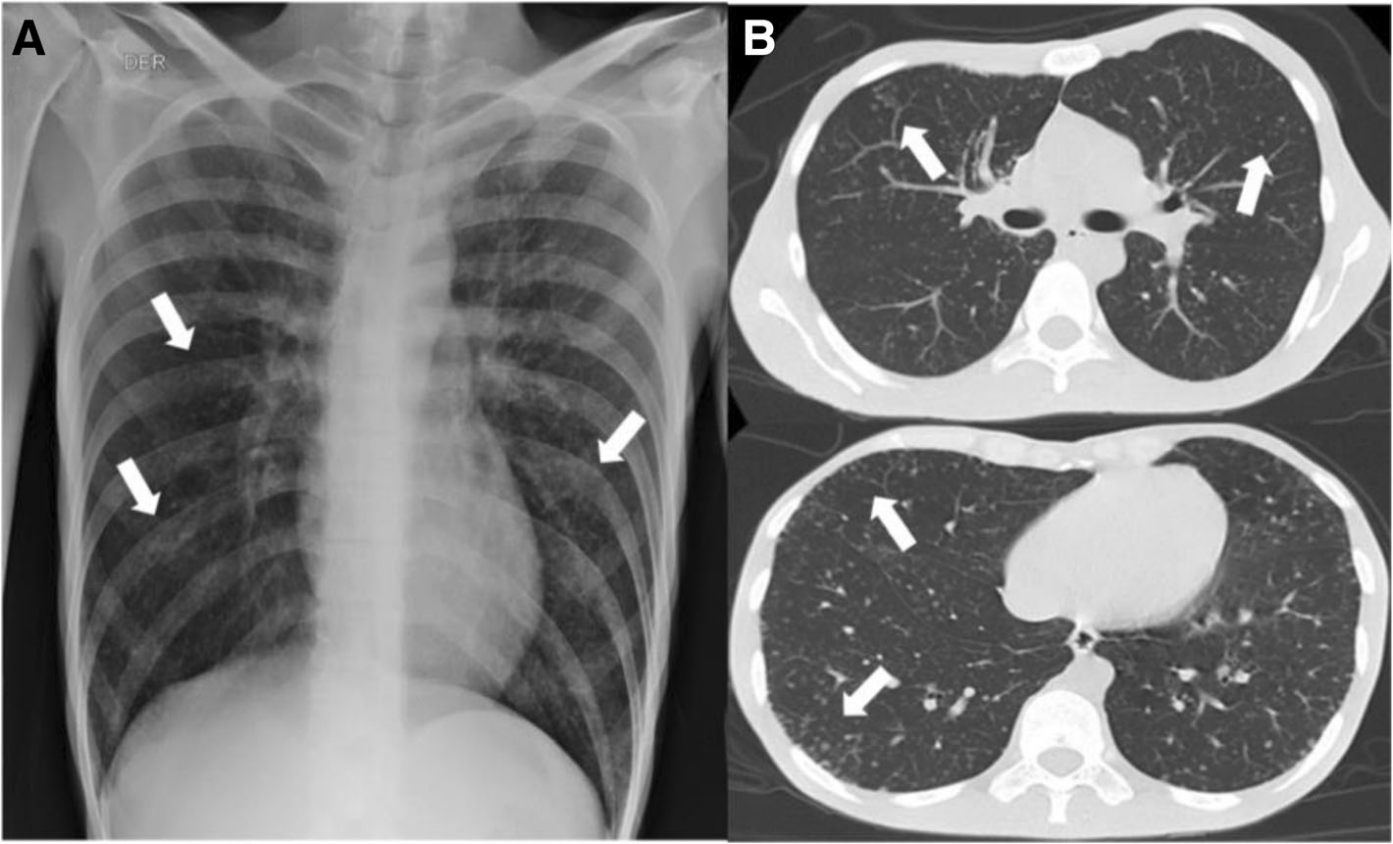
Patient images showing random distribution of micro-nodular opacities. **a**. Chest X-ray. **b**. Thorax CT scan

A chest X-ray and a thorax computed tomography (CT) scan showed a random distribution of micro-nodular lesions, with a preference for peripheral areas of the lungs (Fig. 2). Given these findings, we decided to perform a bronchoscopy with optical fiber with findings of lesions compatible with candidiasis in oropharynx and laryngopharynx, without other lesions present in the rest of the study. The fluid extracted from the bronchoalveolar lavage (BAL) was negative for routine stains: KOH test, Gram and Ziehl Neelsen. Fungi and Mycobacteria cultures and a *GeneXpert* assay for Mycobacterium tuberculosis (MTB) were negative. Cytological studies of the fluid did not exhibit cellular atypia.

Other laboratory tests showed hyperferritinemia (patient value = 1000 ng/mL, reference value 23–336 ng/mL) and mild elevation of alkaline phosphatase (patient value = 152 U/L, reference value = 38–126 U/L) which raised suspicion for histoplasmosis due to the findings of hepatomegaly, skin rash and the random micronodular compromise seen in the chest images. A galactomannan antigen test for *Histoplasma capsulatum* was performed in urine (with enzyme immunoassay (EIA), Immuno Mycologics IMMY) and was positive with 137 ng / mL (reference value < 0.5 ng / ml), which confirmed the diagnosis of disseminated histoplasmosis. Due to these findings, fluconazole was stopped and maintenance therapy was initiated with Itraconazole 200 mg T.I.D for three days and then B.I.D at least for 1 year.

The patient had a satisfactory outcome, with resolution of his diarrhea, disappearance of his rash by the end of the induction phase, and the respiratory symptoms also improved. Patient was discharged with the indication to start HAART with Tenofovir/Emtricitabine 300/200 mg Q.D and Raltegravir 400 mg B.I.D four weeks after induction therapy.

## Discussion

Histoplasmosis and cryptococcosis are two of the fungal diseases with higher prevalence in immunocompromised patients, especially in those with advanced HIV infection and CD4 T lymphocyte cell (CD4) counts lower than 150 and 100 cells/mm3, respectively [7]. Clinical features of both mycoses are similar, presenting with fever, asthenia, adynamia, weight loss, cough, dyspnea, expectoration, diarrhea, lymphadenopathy, hepatomegaly and the beginning of symptoms is insidious [2, 8]. Cutaneous lesions are more frequent in histoplasmosis and the involvement of central nervous system is more frequent in cryptococcosis [3, 4]. Both diseases are causes of morbimortality separately. Report of coinfection of histoplasmosis and cryptococcosis is rare; therefore, a higher mortality can be expected.

In the case presented, we confirmed the diagnosis of two opportunistic infections (Cryptosporidiosis and Cryptococcosis) and we considered the probable diagnosis of histoplasmosis based on the results of the urinary antigen for histoplasmosis and clinical plausibility, mainly for cutaneous manifestations and the random micronodular compromise seen in chest images. In this patient the bronchoalveolar fluid studies were negative and unfortunately no biopsies were taken. There are cross reactions of the urinary antigen test of *Histoplasma capsulatum var. capsulatum* with other infections such as *Paracoccidioides brasiliensis, Blastomyces dermatitidis, Coccidioides immitis*, and *Penicillium marneffei*. These microorganisms should be considered in cases when there are doubts about co-infection with endemic mycoses. [9]. Although it is rare that three opportunistic infections coincide in the same person, our patient had a severe state of immunosuppression and had cutaneous manifestations, which together with the fact that histoplasmosis is endemic in our region, makes a clinically strong plausibility for this infection. We support the diagnosis of Histoplasmosis by the presence of the urinary antigen test of *Histoplasma capsulatum var. capsulatum* that has high sensitivity and specificity and apparently no cross-reaction with cryptococcosis as seen in an antigenuria validation study to diagnose disseminated histoplasmosis in Latin American countries: cross-reactivity was found in three cases of Paracoccidioidomycosis and none in 12 cases of cryptococcosis. [10]. For these reasons, we considered that histoplasmosis was present and therefore, we started antimicrobial treatment with itraconazole as we have already described although we could not have a visual confirmation and considering that cultures have a low diagnostic yield for histoplasmosis.

A Review in PUBMED from 1970 to August/2018 was performed, and 12 cases of patients with coinfection by *Histoplasma capsulatum* and *Cryptococcus spp* were found*.* Other authors performed similar reviews [8, 11]. In Colombia, in a series of 11 cases of Arango et al. [3] a case of coinfection by histoplasmosis and cryptococcosis was reported. So far, we have no information of other cases reported in Colombia. We added both cases to the list of cases reported (Table 1).

**Table 1:** Reported cases of histoplasmosis and cryptococcosis coinfection

	Sex, age, country	Underlying pathology	Treatment	Outcome	Reference
1	Male, 38-years old, United States of America	AIDS	Amphotericin B	Death	[12]
2	Male, 27-years old, United States of America	AIDS	Flucytosine, Fluconazole, trimethoprim/sulfamethoxazole (TMP/SMX)	Alive	[13]
3	Male, 76-years old, Puerto Rico	Diabetes Mellitus, autoimmune thrombocytopenia Corticosteroids	Amphotericin B	Alive	[14]
4	Female, 23-years old, France	AIDS	Amphotericin B, Fluconazole	Alive	[2]
5	Male, 46-years old, Ecuador	AIDS	Amphotericin B	Death	[15]
6	Female, 20-years old, Brazil	AIDS	Amphotericin B, Flucytosine, Fluconazole	Alive	[8]
7	Male, 32-years old, India	AIDS	Amphotericin B, Fluconazole	Alive	[16]
8	Male, 22-years old, Paraguay	AIDS	Amphotericin B, Fluconazole	Alive	[17]
9	Female, 29-years old, Brazil	Immunocompetent	Without treatment	Alive	[18]
10	Male, 27-years old, Ecuador	AIDS	Amphotericin B, Itraconazole	Alive	[19]
11	Male, 69-years old, Brazil	COPD, T2DM	Amphotericin B, Fluconazole	Death	[11]
12	Male, 33-years old, Brazil	AIDS	Amphotericin B, Fluconazole	Alive	[20]
13	1 patient of a case series, Colombia	AIDS	Without information	Without information	[3]
14	Male, 32-years old, Colombia	AIDS	Amphotericin B, Fluconazole, Itraconazole	Alive	Present case

*AIDS* acquired immune deficiency syndrome, *COPD* Chronic Obstructive Pulmonary Disease *T2DM* Type 2 Diabetes Mellitus

There are still regions where HIV detection programs are deficient and facilitate the occurrence of HIV infection cases in advanced stages of immunosuppression. In these patients there must be a high level of suspicion regarding existence of multiple opportunistic infections. We consider that these cases are complex and require an exhaustive search and an adequate correlation with the clinical findings, with the limitation that clinical manifestations can be vague and nonspecific. Cross reactions of non-invasive tests are a concern because, in the case of mycosis many other fungi could be responsible in cross-reactions with the urinary antigen test for histoplasmosis due to the production of galactomannan. But in Latin America this should be considered especially in endemic mycoses such as paracoccidioidomycosis causing cross-reactions with the histoplasmosis antigen test. This essay requires a careful interpretation in these scenarios, but clearly it is an inexpensive, effective and non-invasive tool to reach the diagnosis of histoplasmosis.

## Abbreviations

AIDS: acquired immune deficiency syndrome
BAL: bronchoalveolar lavage
BID: “bis in die”, which means, in Latin, twice a day
BMI: body mass index
CMV: Cytomegalovirus
COPD: Chronic Obstructive Pulmonary Disease
CT: computed tomography
HAART: highly active anti-retroviral therapy
MTB: Mycobacterium tuberculosis
PCR: Polymerase chain reaction
QD: “quaque die”, which means, in Latin, once a day
T2DM: Type 2 Diabetes Mellitus
TID: “ter in die” which means, in Latin, three times a day.
TMP/SMX: Trimethoprim/sulfamethoxazole.
